# Sub-10 fJ/bit radiation-hard nanoelectromechanical non-volatile memory

**DOI:** 10.1038/s41467-023-36076-0

**Published:** 2023-01-28

**Authors:** Yong-Bok Lee, Min-Ho Kang, Pan-Kyu Choi, Su-Hyun Kim, Tae-Soo Kim, So-Young Lee, Jun-Bo Yoon

**Affiliations:** 1 School of Electrical Engineering, Korea Advanced Institute of Science and Technology (KAIST), 291 Daehak-ro, Yuseong-gu Daejeon, 34141 Republic of Korea; 2 National NanoFab Center (NNFC), 291 Daehak-ro, Yuseong-gu Daejeon, 34141 Republic of Korea; 3 Taiwan Semiconductor Manufacturing Company (TSMC) Ltd, Fab 21 Phoenix, AZ USA; 4 SAMSUNG ELECTRONICS Co., Ltd, 1, Samsungjeonja-ro, Hwaseong-si, Gyeonggi-do 18448 Republic of Korea

**Keywords:** Electrical and electronic engineering, NEMS, Mechanical engineering, Electronic devices

## Abstract

With the exponential growth of the semiconductor industry, radiation-hardness has become an indispensable property of memory devices. However, implementation of radiation-hardened semiconductor memory devices inevitably requires various radiation-hardening technologies from the layout level to the system level, and such technologies incur a significant energy overhead. Thus, there is a growing demand for emerging memory devices that are energy-efficient and intrinsically radiation-hard. Here, we report a nanoelectromechanical non-volatile memory (NEM-NVM) with an ultra-low energy consumption and radiation-hardness. To achieve an ultra-low operating energy of less than 10 ${{{{{{\rm{fJ\; bit}}}}}}}^{-1}$, we introduce an out-of-plane electrode configuration and electrothermal erase operation. These approaches enable the NEM-NVM to be programmed with an ultra-low energy of 2.83 ${{{{{{\rm{fJ\; bit}}}}}}}^{-1}$. Furthermore, due to its mechanically operating mechanisms and radiation-robust structural material, the NEM-NVM retains its superb characteristics without radiation-induced degradation such as increased leakage current, threshold voltage shift, and unintended bit-flip even after 1 Mrad irradiation.

## Abstract (web summary)

Achieving both low energy consumption and radiation-hardness is highly challenging in memory devices. Here, the authors demonstrate a sub-10 fJ/bit, radiation-hard nanoelectromechanical non-volatile memory through structural and material approaches.

## Introduction

Conventional semiconductor devices are fundamentally susceptible to radiation due to their material characteristics and operating principles^1^. Three types of radiation-induced damage can occur in semiconductor devices when they are exposed to radiation: total ionization dose (TID)^2^, displacement damage^3^, and single event effects^4^. This damage eventually causes performance degradation in semiconductor memory devices, such as a decreased retention time^5^, threshold voltage shift^6^, and soft error^7,8^. Therefore, in order to protect semiconductor memory devices in harsh radiation environments such as outer space, nuclear power plants, and avionics systems, various radiation-hardening technologies have been investigated from the layout to the circuit and architecture levels^9–12^. However, the application of radiation-hardening technologies involves additional processing and complex circuit configurations that typically incur significant energy overhead^13,14^. For example, to implement the radiation-hard static random-access memory (SRAM) with a TID tolerance of 300 krad (Si), an enclosed gate structure, guard ring, and error detection and correction circuits were additionally introduced. These radiation-hardening processes increase the dynamic power consumption by 1.4 times compared to that of the original^15^. Furthermore, dedicated radiation-hardening processes require larger chip areas and higher production costs compared to the standard process^13^. Therefore, the development of an energy-efficient and intrinsically radiation-hard memory device has become indispensable for next-generation computing. Several studies have reported on the radiation-hardness of magnetic random-access memory (MRAM) and resistive random-access memory (RRAM)^16^. However, radiation-induced damage, such as the deterioration of the tunneling magnetoresistance effect^17^ and the generation of defects in the switching layer^18^, still needs to be improved, respectively.

Nanoelectromechanical non-volatile memory (NEM-NVM) is one of the feasible candidates for radiation-hard electronics applications^19–21^. Thanks to the mechanically operating nature of the NEM device, it has a quasi-zero static power consumption^22^, high on/off ratio^23^, and superb robustness against harsh radiation environments^24^. A conventional NEM-NVM consists of a deformable beam and two in-plane electrodes for the program and erase operations^25^. The deformable beam is deflected laterally due to the electrostatic force applied by each operating in-plane electrode and is held in contact by an adhesion force. The advantage of such a conventional NEM-NVM with an in-plane electrode configuration is that the actuation air gap, deformable beam, and two in-plane electrodes can be fabricated with only one photolithography process (Supplementary Note 1). Due to the ease of manufacturing, the in-plane electrode configuration has been standardized for a long time despite its serious energy efficiency issues^25–29^. The operating energy of the NEM-NVM is greatly affected by the actuation air gap and stiffness ($k{\propto} \left(\right.{w}_{{{{{{{\mathrm{beam}}}}}}}}\,l_{{{{{{{\mathrm{beam}}}}}}}}^{-1}$)3) of the deformable beam, where ${w}_{{{{{{{\mathrm{beam}}}}}}}}$ and ${l}_{{{{{{{\mathrm{beam}}}}}}}}$ are the width and length of the deformable beam^30^. However, the air gap and the width of the conventional NEM-NVM are determined by the resolution of the photolithography tools. Hence, unless the technology node is significantly scaled down to 22 nm or below, the operating energy of the conventional NEM-NVM cannot be reduced to 10 fJ^29^, which is a very challenging level that all emerging non-volatile memory devices are trying to achieve^31^. Furthermore, the electrostatic erase operation inevitably doubles the air gap distance, resulting in a high-energy consumption and operating voltage starting with the second operation^25^.

To overcome such limitations related to the operating energy of the NEM-NVM, a sub-10 nm air gap formation method with an electroburning process of single-walled carbon nanotubes (SWNTs) was proposed recently^32^. The fabricated SWNT memory device has an ultra-low energy consumption of less than 1 ${{{{{{\rm{aJ\; bit}}}}}}}^{-1}$. However, the electrostatic erase operation significantly increased the actuation air gap, resulting in a high operating energy and voltage. More recently, a quad electrode architecture was introduced to maintain the actuation air gap during operation^33^. However, the large initial air gap (120 nm) due to the in-plane electrode configuration is still a major cause of hindering the energy-efficient operation.

Here, we report a sub-10 ${{{{{{\rm{fJ\; bit}}}}}}}^{-1}$ energy-efficient and radiation-hard NEM-NVM fabricated with well-established complementary metal–oxide–semiconductor (CMOS) manufacturing processes. The ultra-low energy consumption of the NEM-NVM is achieved through two strategies: an out-of-plane electrode configuration and an electrothermal erase operation. The out-of-plane electrode configuration enables efficient scaling of the actuation air gap and stiffness through the film deposition process, regardless of the resolution of the lithography tools. Additionally, the electrothermal erase operation recovers the programmed beam to its initial state without increasing the actuation air gap distance. As a result, we successfully demonstrate an energy-efficient NEM-NVM with an ultra-low programming energy of 2.83 ${{{{{{\rm{fJ\; bit}}}}}}}^{-1}$. Furthermore, the NEM-NVM shows radiation-hardness against ^60^Co gamma irradiation with a total dose of 1 Mrad, which is the tolerance of radiation-hardened silicon-based electronics.

## Results

### Device architecture and operation mechanisms

**Fig. 1:**
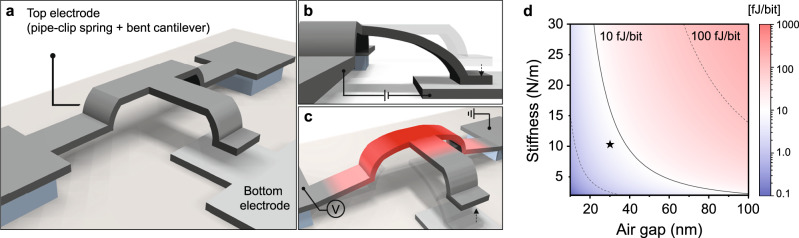
Structure, operation mechanisms, and calculated programming energy of the nanoelectromechanical non-volatile memory (NEM-NVM). **a** Schematic of the NEM-NVM with an out-of-plane electrode configuration. **b** Schematic illustration of the program operation. The program operation is driven by electrostatic force due to the voltage difference between the top and bottom electrodes, and the adhered bent cantilever is maintained by the intentionally designed adhesion force. **c** Schematic illustration of the erase operation. The erase operation is driven by electrothermal force due to the resistive heating of the pipe-clip spring, and the adhered bent cantilever is recovered to its initial state. **d** Calculated programming energy of the NEM-NVM with respect to the actuation air gap and stiffness of the top electrode. To realize a programming energy of less than 10 ${{{{{{\rm{fJ\; bit}}}}}}}^{-1}$, the air gap and stiffness were designed to be 30 nm and 10.15 ${{{{{{\rm{N\; m}}}}}}}^{-1}$, respectively (marked by a star).

Figure 1a shows a schematic image of the NEM-NVM with an out-of-plane electrode configuration. The top and bottom electrodes are arranged vertically each other, and they are completely separated by an actuation air gap. The bent cantilever and pipe-clip spring of the top electrode are delicately designed to achieve low programming energy. The bent cantilever enables a small air gap between the top and bottom electrodes, and the pipe-clip spring is devised for an electrothermal erase operation that recovers the programmed bent cantilever to its initial state. In the initial state of the NEM-NVM (high contact resistance state, HCRS), it has an ultra-low off-state current of less than 100 fA due to the physically separated gap. Figure 1b shows the program operation. When an operating voltage is applied between the top and bottom electrodes, the bent cantilever is deflected in the vertical direction due to the electrostatic force, which establishes a current path between the top and bottom electrodes. If the adhesion force between the bent cantilever and the bottom electrode is designed to be larger than the restoring force of the deflected bent cantilever, the adhered state is maintained even if the applied voltage is switched off (low contact resistance state, LCRS). In other words, the stiction phenomenon is intentionally utilized to obtain non-volatility. Unlike the conventional NEM-NVM with an in-plane electrode configuration, for which it is difficult to make conformal contact due to inclined sidewalls, the proposed out-of-plane electrode structure enables conformal contact between the top and bottom electrodes because of the flat contacting surfaces. Through a three-dimensional finite-element method (FEM) simulation of the program operation, we confirmed that the bent cantilever made conformal contact at the contacting surfaces (Supplementary Note 2), which can contribute to improving the contact characteristics such as stable retention performance. Figure 1c shows the erase operation of the NEM-NVM. When an operating voltage is applied across the ends of the pipe-clip spring of the top electrode, resistive heating begins to induce a temperature increase, resulting in mechanical thermal expansion in the upward direction. If the generated thermal expansion force is sufficiently greater than the adhesion force, the adhered bent cantilever is detached from the bottom electrode. Unlike the conventional electrostatic erase operation, the electrothermal erase operation recovers the programmed bent cantilever to its initial state. Figure 1d shows the calculated programming energy of the NEM-NVM as a function of the air gap and stiffness of the top electrode (a detailed calculation process is provided in Supplementary Note 3). To realize a programming energy of less than 10 ${{{{{{\rm{fJ\; bit}}}}}}}^{-1}$, the air gap (${g}_{{{{{{{\mathrm{air}}}}}}}}$) and stiffness ($k$) were determined to be 30 nm and 10.15 ${{{{{{\rm{N\; m}}}}}}}^{-1}$, respectively (starred in Fig. 1d). These two parameters were carefully considered along with the mechanical analysis for the stable program and erase operations as they also have a great influence on the operations of the device.

### Mechanical analysis for the stable program and erase operations

In order for the NEM-NVM to behave in a non-volatile manner in the program operation, the adhesion force (${F}_{{{{{{{\mathrm{adh}}}}}}}}$) between the contacting electrodes must be designed to be larger than the restoring force (${F}_{r}={g}_{{{{{{{\mathrm{air}}}}}}}}\times k$) of the deflected beam. Therefore, accurate analysis of the adhesion force and restoring force, respectively, is critical in the mechanical design of the NEM-NVM. First, we calculated the adhesion force between tungsten surfaces using the well-established previous adhesion model^34^. The detailed analysis process of the adhesion force is shown in Supplementary Note 4. To model the physical interactions across the interface that we need to analyze, actual surface roughness data should be reflected. Therefore, the surface roughness of the samples having the same thickness as the top and bottom electrodes was measured using an atomic force microscope (AFM), respectively. And then, the measured surface roughness data were imported into the adhesion model to mimic the actual contact process. In this contact process, we considered the two dominant forces between the metallic surfaces to calculate an accurate adhesion force: metallic bonding force between the contacting asperities and van der Waals force across the non-contacting areas^35^. Consequently, the final adhesion force in the area of 0.03 $\,{{{{{{\rm{\mu }}}}}}{{{{{\rm{m}}}}}}}^{2}$, which is the overlap area between the top and bottom electrodes, was determined to be 0.96 ${{{{{\rm{\mu }}}}}}$N. Next, for analysis of the restoring force, the stiffness ($k$) of the complex top electrode should be calculated accurately. Thus, we used Castigliano’s theorem in conjunction with the principle of virtual force to model the stiffness of the NEM-NVM as shown in Supplementary Fig. 4. The bending moments and torsions by the virtual force at each simplified element of the top electrode were found, and the displacement and stiffness at the end of the bent cantilever were calculated using Castigliano’s theorem. Supplementary Fig. 5a shows the restoring force with respect to the length of the bent cantilever and the length of the pipe-clip spring. The NEM-NVM was designed in the range of the blue region, which ensures the non-volatility to achieve the stable LCRS even if the program voltage is switched off.

In the case of the erase operation, the thermal expansion force must be greater than the adhesion force. To calculate the thermal expansion force, we conducted an electrothermal FEM simulation to derive the temperature profile of the pipe-clip spring (Supplementary Fig. 5b) and applied it to the thermo-mechanical model^36^. Supplementary Fig. 5c shows the calculated thermal expansion force with respect to the length of the pipe-clip spring and operating temperature. These results show that the shorter the pipe-clip spring, the lower the erasing temperature. However, the short-length pipe-clip spring can cause unintended erase operation in high-temperature environments. Therefore, the length of the pipe-clip spring was determined to be 2200 nm so that the NEM-NVM could achieve high stability of the mechanical bit even in the high-temperature environment up to 400 K, which is the required temperature range for practical applications^37^. Moreover, the compressive thermal stress generated in the pipe-clip spring during the erase operation was expected to be 1.5 GPa through the FEM simulation, which is lower than the yield stress of tungsten nanostructure (~2 GPa)^38^. Finally, the dimension information of the optimally designed NEM-NVM is shown in Supplementary Fig. 6.

### CMOS-compatible device fabrication on an 8-inch wafer

**Fig. 2:**
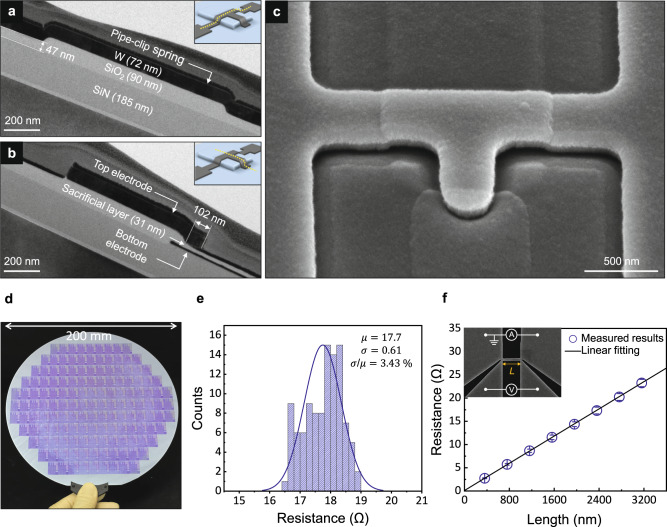
CMOS-compatible device fabrication. **a**, **b** Cross-sectional TEM images of the pipe-clip spring and bent cantilever of the fabricated NEM-NVM, respectively. Inset figures indicate the cross-section plane. **c** Tilted-view SEM image of the air-suspended NEM-NVM after the release process. **d** Optical photograph of the 8-inch Si wafer fabricated by well-established CMOS manufacturing processes. **e** Large-area uniformity of the resistance of the pipe-clip springs represented by histograms (105 devices). $\mu$ and $\sigma$ represent the average and standard deviation of the resistance, respectively. **f** Measured electrical resistance with respect to the length (*L*) of the fixed-fixed tungsten beam. The fixed-fixed tungsten beam with the 4-point probe electrodes is shown in the inset (width, $W$ = 300 nm). The error bars represent the standard deviations among the beams (*n* ≥ 30).

Considering reliable fabrication and compatibility with standard CMOS architectures, selecting the fabrication processes and structural materials is an important step. We utilized the well-established CMOS manufacturing processes to fabricate the NEM-NVM on an 8-inch wafer (detailed fabrication steps are provided in Methods and Supplementary Note 6). The selected structural material is tungsten, which is robust to high-energy photons and particles due to its radiation shielding property and low sputtering yield^39,40^. Moreover, it has already been used as a back-end-of-line material^41^. After the fabrication processes, cross-sectional transmission electron microscope (TEM) images were obtained to confirm the thickness of the top electrode and sacrificial layer, and the step height of the pipe-clip spring, which have a great influence on the operation of the NEM-NVM. Figure 2a, b present cross-sectional TEM images of the pipe-clip spring and bent cantilever of the fabricated NEM-NVM before the release process, respectively. Through these results, we confirmed that the important parameters, such as the thickness of the top electrode and sacrificial layer, were well fabricated and matched the optimum design, as shown in Supplementary Fig. 6. Figure 2c shows a scanning electron microscope (SEM) image of the air-suspended NEM-NVM after removing the sacrificial layer using the buffered oxide etchant (BOE). To prevent unintended initial stiction, a critical point dryer process was also used. Unlike the conventional NEM memory devices, the fabricated NEM-NVM does not require long-cantilever and in-plane electrodes, which made it possible to have a small footprint of 21 F^2^ (F stands for feature size). A large number of devices were fabricated on an 8-inch wafer through the reliable CMOS manufacturing processes (Fig. 2d). To evaluate the uniformity of the pipe-clip springs, we measured the electrical resistance of the pipe-clip springs from 105 different devices using the four-point probe method. The reliable fabrication processes enabled a high spatial uniformity of 3.43 % in a coefficient of variation as shown in Fig. 2e. As the dimensions of the device are scaled, the electrical and thermal properties of metals are changed due to the size-dependent electron-phonon effect^42,43^. Therefore, to analyze the operating characteristics of the fabricated NEM-NVM, it is essential to evaluate the electrical and thermal conductivity of nano-patterned tungsten. Figure 2f shows the measured electrical resistance with respect to the length of a fixed-fixed tungsten beam. The experimentally determined electrical conductivity ($\sigma$) was 5.75 $\times {10}^{6}\,{{{{{{\rm{S\; m}}}}}}}^{-1}$, and the thermal conductivity ($\kappa$) was determined from the Wiedemann–Franz law (${LT}=\kappa \,{\sigma }^{-1}$), where *L* is the Lorenz number and *T* is the absolute temperature. The calculated thermal conductivity (47.9 ${{{{{\rm{W}}}}}}{{{{{{\rm{m}}}}}}}^{-1}\,{{{{{{\rm{K}}}}}}}^{-1}$) was reduced by 71.8% compared to that of bulk material (170 ${{{{{\rm{W}}}}}}{{{{{{\rm{m}}}}}}}^{-1}\,{{{{{{\rm{K}}}}}}}^{-1}$)^44^. The reduced thermal conductivity induced heat confinement in the pipe-clip spring during the joule heating process, which enabled a fast and energy-efficient to erase operation^45^.

### Electrical and optical evaluations

**Fig. 3:**
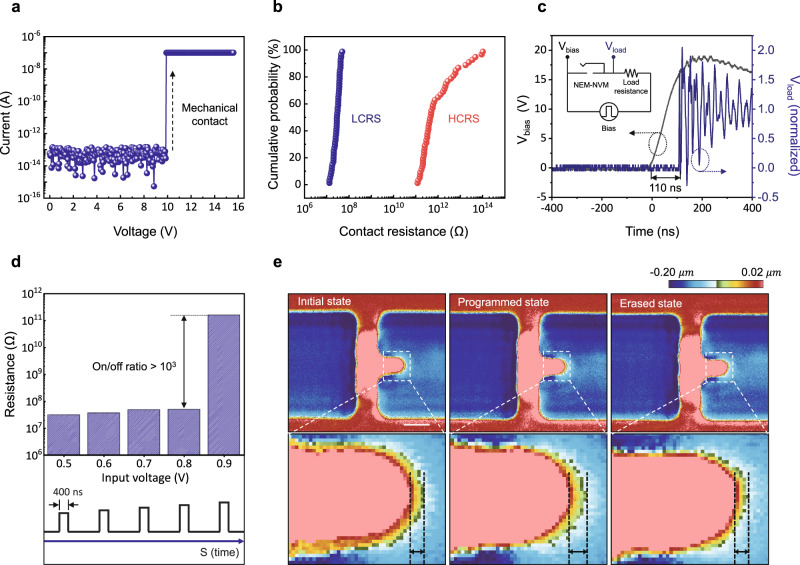
Electrical and optical evaluations. **a** Current–voltage (I–V) characteristics of the fabricated NEM-NVM. At the sub-threshold region (${{{{{\rm{V}}}}}}$< ${{{{{{\rm{V}}}}}}}_{{{{{{\rm{program}}}}}}}$), an ultralow off-state current of less than 100 fA was observed. The current curve increased abruptly at an applied voltage of 9.9 V (compliance current of 100 nA). **b** Contact resistance distribution represented by cumulative probability (total 100 devices). The blue circle represents the low contact resistance state (LCRS) of the programmed device, and the red circle represents the high contact resistance state (HCRS) of the unprogrammed device. The contact resistance was measured through the four-point probe method at one-half programming voltage with 100 nA compliance current. **c** Evaluation of the programming time. The voltage divider circuit is shown in the inset. The black line represents the bias pulse, and the blue line represents the divided voltage to the load resistor. The time difference between the bias pulse and divided voltage is the programming time (<110 ns). **d** Contact resistance with respect to the application of the voltage pulse. When an erase voltage pulse (0.9 V, 400 ns) was applied, the contact resistance increased abruptly, indicating a successful erase operation. **e** Surface profile images of three states (initial, programmed, and erased states) measured by high-resolution 3-dimensional laser scanning confocal microscope (scale bar = 500 nm).

We conducted electrical/optical measurements to verify the operations of the fabricated NEM-NVM. All electrical measurements were performed under vacuum conditions (<1 mTorr). Fig 3a shows the current–voltage (I−V) characteristics of the fabricated NEM-NVM. A noise-level current (~100 fA) was observed in the sub-threshold region due to the physically separated gap between the top and bottom electrodes. This low off-current allows the NEM-NVM to have quasi-zero static power consumption. When the applied voltage exceeded the threshold (program) voltage of 9.9 V, the memory exhibited an abrupt switching characteristic, indicating that mechanical contact was made. The calculated sub-threshold swing (SS) was less than 10 mV dec^−1^, which was much smaller than the theoretical limit (60 mV dec^−1^) of the typical semiconductor devices. To calculate the exact programming energy, the actual air gap of the NEM-NVM needs to be estimated. Using the measured programming voltage (9.9 V), the actual air gap of the NEM-NVM was estimated to be about 20 nm through the analytical stiffness model and 3D FEM simulation (Supplementary Fig. 8a). As a result, the programming energy of the fabricated NEM-NVM was calculated to be 2.83 ${{{{{\mathrm{f}}}}}}{{{{{\mathrm{J}}}}}}\,{{{{{\mathrm{bit}}}}}}^{-1}$, which is almost 30,000 times smaller than that of non-volatile flash memory^31^. We also confirmed that the fabricated NEM-NVM operated in a non-volatile manner through the I–V dual sweep curves (Supplementary Fig. 8b). Figure 3b shows the cumulative probability of the LCRS and HCRS. This result demonstrates a small device-to-device variation of LCRS and a high on/off ratio (>10^3^). The contact resistance was measured through the four-point probe method. The transient switching response of the NEM-NVM was also measured through the voltage divider circuit plotted in Fig. 3c. The applied pulse is divided into the load resistor from the moment the NEM-NVM is switched on. In other words, the time taken for the applied voltage to divide into the load resistor is the programming time of the NEM-NVM. The measured programming time was less than 110 ns, which is faster than those of any NEM memory devices researched so far^23,29,33^. The occurrence of mechanical contact can also be confirmed by an underdamped response. The resonance frequency extracted from the underdamped response (40.5 MHz) was similar to that of the FEM simulation result (41.9 MHz, Supplementary Fig. 12b). Next, for the erase operation, a voltage pulse was applied across the ends of the pipe-clip spring of the programmed device. When a voltage pulse of 0.9 V for a time duration of 400 ns was applied, the contact resistance increased abruptly, indicating a successful erase operation (Fig. 3d). This fast and low voltage electrothermal erase operation was attributed to an extremely small heat capacity (0.157 ${{{{{\rm{pJ}}}}}}$ K^−1^) and an enhanced thermal concentration associated with the reduced thermal conductivity (47.9 ${{{{{\rm{W}}}}}}{{{{{{\rm{m}}}}}}}^{-1}$ K^−1^). The erase temperature can be estimated using the resistance temperature detector (RTD) method and time-dependent FEM simulation (Supplementary Note 8). The evaluated maximum erase temperature was approximately 873.1 K. In addition, since the NEM-NVM operates mechanically, the state of the bent cantilever at each state can be visually observed. Figure 3e shows the surface profile images of the single device at released, programmed, and erased states, which were measured by a high-resolution three-dimensional laser scanning confocal microscope (keyence VK-250). The surface profile image of the released state reveals that the fabricated NEM-NVM was perfectly suspended without stiction. After the program operation, the cantilever was bent in the vertical direction and maintained an adhered state to the bottom electrode even when the actuation voltage was switched off, as shown in the programmed state. Finally, after the erase operation, the bent cantilever was recovered to its initial state. Actually, it is difficult to distinguish each state since the bent cantilever has a very small range of motion (about 20 nm). Nevertheless, these results are very meaningful in that the mechanical behavior of the NEM-NVM with an out-of-plane electrode configuration was optically evaluated for the first time. Also, these are direct evidence that the NEM-NVM operated successfully according to the proposed operating methods.

**Fig. 4:**
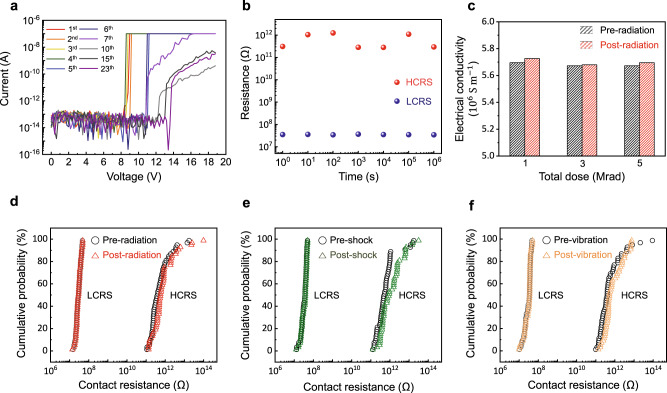
Basic NEM-NVM performance and robustness of mechanical bits. **a** Current–voltage (I–V) characteristics during cyclic measurements. **b** Stable retention behavior for a time of 10^6^ s at room temperature. **c** Electrical conductivity of nano-patterned tungsten before and after exposure to various total radiation doses (1, 3, and 5 Mrad). **d** Gamma-radiation stability (1 Mrad) of the mechanical bits. The black circle represents LCRS and HCRS before exposure to gamma-radiation, and the red triangle represents LCRS and HCRS of the same devices after 1 Mrad irradiation (100 devices). **e**, **f** Mechanical shock (2900 G) and vibration (2000 Hz) stability of the mechanical bits, respectively. Equally, the LCRS and HCRS were evaluated before and after the mechanical shock and vibration experiments (100 devices).

Figure 4a presents the I–V characteristics during cyclic measurements and shows an endurance of >20 cycles. The initial few I–V curves of the repetitive programming operations show that the NEM-NVM was programmed at very similar actuation voltages ranging from 8.6 to 9.2 V. This electrical result means that the fabricated NEM-NVM had the same actuation air gap distance during the repetitive programming operations, unlike the conventional NEM-NVM. Also, it is further evidence that the adhered bent cantilever was recovered to its initial state through the electrothermal erase operation. However, two problems, unstable current characteristics and increased operating voltages during endurance tests, need to be improved. The unstable current characteristics can be caused by physical degradation at the contacting surfaces, such as mechanical wear, material transfer, and electrical arcing^46^. Therefore, further research is needed to introduce the superb contact materials that have already been proven to improve the reliability of micro-contact switches. For example, introducing a Ru^47^, Pt^48^, graphene^49^, CNT^50^, and alloys^51^ as contact materials at the interface can improve mechanical wear and material transfer. Also, an ultra-thin SiO_2_ layer^52^ (~8 nm) as the contact materials can help to alleviate the electrical arcing or excessive joule heating at the contacting surfaces. The second problem is the increased operating voltage during the endurance test. The erase operation of the NEN-NVM is driven by the electrothermal method. If the generated thermal stress during the erase operation is greater than the yield stress, plastic deformation occurs in the pipe-clip spring, which can lead to an increase in the actuation air gap and operating voltage. As we described in the mechanical analysis section (mechanical analysis for the stable program and erase operations), the expected maximum temperature and compressive thermal stress during the erase operation were about 700 K and 1.5 GPa, respectively. However, the actual maximum temperature was about 870 K, and the compressive thermal stress at the actual temperature was about 2.3 GPa, which is higher than the yield stress of tungsten nanostructure^38^. The reason why the actual erase temperature was higher than the designed temperature is that the actual adhesion force was larger than the predicted adhesion force. When we predicted the adhesion force, roughness data of the surfaces having the same thickness as the top and bottom electrodes of the NEM-NVM were used because the actual contacting surfaces are buried in the bodies. This analysis process may lead to an error in the prediction of the adhesion force. There are two solutions to improve the plastic deformation issues for high endurance. The first one is to use N pipe-clip springs. If N pipe-clip springs are used, the same erase force can be generated even at a lower operating temperature because the stiffness of the thermal actuator part is increased N times. The other is to use a cantilever-shaped thermally actuated structure^53^. In general, a cantilever beam is free from thermal stress as it can be stretched freely in length. Therefore, the two proposed methods are expected to be promising research topics for improved endurance of the NEM-NVM by suppressing fatigue and plastic deformation. Figure 4b shows the highly stable retention characteristics of the NEM-NVM over 10^6^ s at room temperature. This excellent data retention while retaining a high on/off ratio (>10^3^) is due to the flat contacting interface and physically separated gap.

### Stability of the mechanical bits in harsh environments

The optimally designed mechanical operations, robust material, and small mass of the nanostructure allow the NEM-NVM to retain the stored mechanical bits after exposure to high-energy radiation as well as shock, vibration, and high temperature. First, the electrical conductivity of nano-patterned tungsten, which is the same structure as the inset in Fig. 2f, was evaluated after exposure to various total radiation doses (1, 3, and 5 Mrad) to confirm the radiation effects on the structural material of the fabricated NEM-NVM. As shown in Fig. 4c, the differences in the electrical conductivity before and after the gamma-ray irradiation in each condition were very small, 0.54, 0.27, and 0.40%, respectively (detailed electrical measurement results are provided in Supplementary Fig. 10). This excellent robustness is due to the radiation shielding property of tungsten. Figure 4d shows the cumulative probability of the HCRS and LCRS of the NEM-NVM before and after the 1 Mrad gamma-ray irradiation, which is the tolerance of radiation-hardened electronics^54^. All 100 devices retained their original HCRS and LCRS without unintended bit-flip, and the distributions of the LCRS before and after irradiation are almost the same. The high contact resistance of the unprogrammed device is determined by the noise current, so the distribution of HCRS changes with each measurement. Furthermore, the I–V characteristics of the NEM-NVM exposed to radiation were also measured. As shown in Supplementary Fig. 11, the characteristics of the exposed device, namely, ultra-low leakage current, operating voltage, and steep sub-threshold swing, were almost identical to those of the unexposed device. This superb radiation-hardness of the NEM-NVM is due to its mechanically operating mechanisms and radiation-robust structural material.

Since the NEM-NVM has a physically separated gap, there may be concerns about the stability of stored information when it is exposed to external stimuli such as mechanical shock and vibration. However, the NEM-NVM is expected to be insensitive to mechanical shock and vibration due to its ultra-small mass. The force ($F$) exerted on a proof mass by acceleration, and the resulting displacement ($\delta$) can be calculated through the simple equations ($F=$ *ma* and $\delta=F\,{k}^{-1}$, where *m* is a proof mass, *a* is the acceleration, and $k$ is the stiffness)^55^. The mass of the top electrode of the NEM-NVM is $1.18\times {10}^{-15}$ kg. The exerted force, even at high acceleration, is very small, so the resulting displacement of the NEM-NVM is negligible compared to the actuation air gap, as shown in Supplementary Fig. 12a. The ultra-small mass is also the main reason for the high resonance frequency (${{{{{\rm{\omega }}}}}}\propto \sqrt{k{m}^{-1}}$) of the fabricated NEM-NVM. The resonance frequency was confirmed to be 41.9 MHz through the FEM simulation (Supplementary Fig. 12b), and it was also confirmed experimentally as discussed in Fig. 3c. The high resonance frequency ensures robustness against vibrations in general applications. Figure  4e, f show the cumulative probability of the HCRS and LCRS of the NEM-NVM before and after exposure to mechanical shock and vibration, respectively. The experimental conditions were prepared based on the Joint Electron Device Engineering Council (JEDEC) standard (see Methods). As expected theoretically, all devices retained their HCRS and LCRS without unintended bit-flip, and the distributions of the LCRS before and after the mechanical shock and vibration are almost the same. The excellent stability of the mechanical bits against external stimuli was experimentally verified for the first time. The NEM-NVM was also designed to have high stability in ambient temperature. Therefore, the displacement of the bent cantilever over the ambient temperature range from 233 to 378 K is less than 4 nm, as shown in Supplementary Fig. 13a. This result indicates that unintended program operation by the ambient temperature cannot occur. Meanwhile, the programming voltage may shift as the actuation air gap changes due to the temperature-induced displacement of the bent cantilever. However, the voltage shift in this temperature range is less than ±2 V. Undesired erase operation in high-temperature environments was also an important consideration. Thus, the NEM-NVM was designed so that the generated thermal force in high-temperature environments is much smaller than the adhesion force even when the ambient temperature is increased to 480 K, which ensures the high stability of the mechanical bit in high-temperature environments (Supplementary Fig. 13b). To evaluate the stability of the mechanical bit, we demonstrated the superb stability of both HCRS and LCRS at high temperature up to 400 K as shown in Supplementary Fig. 13c. These experimental results prove that the NEM-NVM is highly robust against not only high-energy radiation but also shock, vibration, and high temperature. Table 1 presents a comparison of the recently reported NEM-NVM devices related to our work. Our NEM-NVM has a very small actuation air gap and ultra-low programming energy even though it was fabricated with the conventional 300 nm CMOS manufacturing processes. This result indicates that a highly energy-efficient NEM-NVM can be achieved through the out-of-electrode configuration and delicate structural design without the use of advanced lithography tools or special materials and processes. The key parameters of the memory device, such as the footprint, programming time, and retention characteristics, also show superb performance compared to those of the previous studies. Furthermore, while a few other studies have reported the stability of the mechanical bits only at high temperatures, our NEM-NVM shows excellent stability of the mechanical bits in various harsh environments, including high-energy radiation.

**Table 1:** Comparison of NEM-NVM devices

Reference	^29^	^32^	^33^	^23^	This work
Electrode configuration	In-plane	In-plane	In-plane	Out-of-plane	Out-of-plane
Operating mechanisms	Electrostatic program & erase	Electrostatic program & erase	Electrostatic program & erase	Electrostatic program & erase	Electrostatic program Electrothermal erase
Footprint	$\sim$ 6.5 ${{{{{\rm{\mu }}}}}}$m $\times$ 0.4 ${{{{{\rm{\mu }}}}}}$m	Single wall CNT	$\sim$ 10 ${{{{{\rm{\mu }}}}}}$m $\times$ 5 ${{{{{\rm{\mu }}}}}}$m	$\sim$ 8 ${{{{{\rm{\mu }}}}}}$m $\times$ 1.5 ${{{{{\rm{\mu }}}}}}$m	2.2 ${{{{{\rm{\mu }}}}}}$m $\times$ 0.9 ${{{{{\rm{\mu }}}}}}$m
Actuation air gap	230 nm	<5 nm	120 nm	500 nm	20 nm
Fabrication method	BEOL CMOS manufacturing processes (65 nm node)	Electroburning method & electron beam lithography	Electron beam lithography	Electron beam lithography	KrF scanner (300 nm node)
Structural material	Cu, TiN, TaN	CNT	Si, Ti	Cr, Al, Au	W
Programming voltage	9.75 V	1.7 V	>7 V	16 V	>8.6 V
Programming energy	>80 ${{{{{{\rm{fJ\; bit}}}}}}}^{-1}$ (simulated)	0.41 ${{{{{{\rm{aJ\; bit}}}}}}}^{-1}$			2.83 ${{{{{{\rm{fJ\; bit}}}}}}}^{-1}$
Programming time	>400 ns (simulated)		511 ns (simulated)	130 ns	110 ns
Erase voltage		21 V	>8 V	16 V	0.9 V
Retention		40 h	>6 months	41,000 s	>10^6^ s
Endurance	>3 cycles	10 cycles	42 cycles	500 cycles	23 cycles
Robustness in harsh environments			High temperature (473 K)		Radiation (1 Mrad), shock (2900 G), vibration (2000 Hz), high temperature (400 K)

## Discussion

We have demonstrated a NEM-NVM that achieves both an ultra-low programming energy of 2.83 ${{{{{{\rm{fJ\; bit}}}}}}}^{-1}$and radiation-hardness against ^60^Co gamma irradiation with a total dose of 1 Mrad. To overcome the operating energy issues of the conventional NEM-NVM, an out-of-plane electrode configuration and electrothermal erase operation are introduced. The out-of-plane electrode configuration enables efficient scaling of the air gap and stiffness, the most important factors of the programming energy. Moreover, the electrothermal erase operation successfully recovers the adhered bent cantilever to its initial state, which is corroborated by high-resolution 3D surface profiler results and electrical evaluations. As a result, we demonstrate an energy-efficient NEM-NVM with programming energy of less than 10 ${{{{{\rm{fJ}}}}}}\,{{{{{{\rm{bit}}}}}}}^{-1}$. Furthermore, unlike the semiconductor memory devices, the fabricated NEM-NVM can retain its superb characteristics without radiation-induced performance degradation, including an increased leakage current, threshold voltage shift, and unintended bit-flip even after exposure to 1 Mrad radiation. The stability of the stored mechanical bits against mechanical shock (2900 G), vibration (2000 Hz), and high temperature (400 K) is also demonstrated. Our successful demonstration and the unprecedented superior performance of the NEM-NVM will significantly open new possibilities for future applications that require both high-energy efficiency and radiation-hardness.

## Methods

### Device fabrication

The NEM-NVM was fabricated using the well-established CMOS manufacturing processes on an 8-inch silicon wafer. First, a silicon nitride (200 nm thick) was deposited on a thermally oxidized silicon wafer. For the formation of the bottom electrode, tungsten (15 nm thick) was deposited and then patterned by the conventional photolithography process. In order to make a sophisticated step profile for the pipe-clip structure, a polishing process is needed to make a flat surface. Therefore, silicon dioxide (200 nm thick) was deposited via plasma-enhanced chemical vapor deposition (PECVD) equipment, and chemical-mechanical polishing (CMP) was used. The bottom tungsten electrode serves as a stopping layer in the CMP process. Then, a second silicon dioxide (20 nm thick) was deposited and patterned to form the pipe-clip structure. A third silicon dioxide (30 nm thick) was deposited, which serves as an actuation air gap between the top and bottom electrodes. For the formation of the top electrode, a sputtering deposition method was used to make the top electrode follow the surface profile of the patterned sacrificial layer. Since sputtered tungsten has residual stress, an annealing process is needed to alleviate it. Therefore, the NEM-NVM was passivated by silicon dioxide (100 nm thick) and then annealed in a vacuum furnace (500 °C, 30 min). Finally, the sacrificial silicon dioxide was etched using a BOE solution, and critical point drying (CPD) was used to dry the devices without stiction. (Schematic images of the overall fabrication process are provided in Supplementary Note 6)

### Electrical measurements

The fabricated NEM-NVM was measured under a vacuum probe station (MSTECH, vacuum chamber M6VC). The current–voltage characteristic was measured using the Keithley 2636B parameter analyzer, and the contact resistance was measured through the four-point probe method using the Keithley 2636B parameter analyzer and Keithley 2182 A nanovoltmeter with one-half programming voltage and a 100 nA compliance current. The programming time was measured using the function generator (Tektronix, AFG31252) and Oscilloscope (Tektronix, MSO44 4-BW-500).

### Harsh environment experiments

Gamma-ray irradiation was performed at the Advanced Radiation Technology Institute in South Korea. The NEM-NVM was exposed to ^60^Co gamma-ray radiation with a maximum total dose of 1 Mrad. Mechanical shock and vibration experiments were performed at the QRT in South Korea, a company that specializes in reliability testing. The experimental conditions of mechanical shock and vibration were prepared based on the JEDEC standard. The conditions for the mechanical shock test were as follows: acceleration peak (2900 G), pulse duration (0.3 ms), equivalent drop height (150 cm), and velocity change (543 cm/s). The conditions for the vibration test are as follows: min./max. frequency (20/2000 Hz), peak acceleration (20 G), displacement pk-pk (1.5 mm), and cross-over frequency (80 Hz). The high-temperature experiment was performed under a vacuum probe station with a hot chuck controller (MSTECH, MST 1000B).

## Supplementary information

Supplementary information

## Data Availability

All data are available within the Article and its Supplementary Information files. Additional information is also available from the corresponding author upon reasonable request.

## Code availability

All codes used for calculations and data analysis are available from the corresponding author upon reasonable request.
