# Supplementary material for: Sub-10 fJ/bit radiation-hard nanoelectromechanical non-volatile memory
Source: Nat Commun. 2023 Jan 28;14:460. doi: 10.1038/s41467-023-36076-0 (PMC9884203; doi:10.1038/s41467-023-36076-0)
Supplement: Supplementary file 1 — Supplementary information [file 41467_2023_36076_MOESM1_ESM.pdf]

Supplementary Information for:

Sub-10 fJ/bit Radiation-hard Nanoelectromechanical Non-volatile Memory

*Yong-Bok Lee[1], Min-Ho Kang[2], Pan-Kyu Choi[1,3], Su-Hyun Kim[1,4], Tae-Soo Kim[1], So-Young Lee[1] and Jun-Bo Yoon[1]**

[1]School of Electrical Engineering, Korea Advanced Institute of Science and Technology (KAIST), 291 Daehak-ro, Yuseong-gu, Daejeon 34141, Republic of Korea

[2]National NanoFab Center (NNFC), 291 Daehak-ro, Yuseong-gu, Daejeon 34141, Republic of Korea

[3]Taiwan Semiconductor Manufacturing Company (TSMC) Ltd. Fab 21, Phoenix, Arizona, USA.

[4]SAMSUNG ELECTRONICS Co., Ltd. 1, Samsungjeonja-ro, Hwaseong-si, Gyeonggi-do 18448, Republic of Korea

*Corresponding author: Jun-Bo Yoon

Tel: +82-42-350-3476, Fax: +82-42-350-8565, E-mail: jbyoon@kaist.ac.kr

Table of contents

Supplementary Note 1: Limitations of the conventional NEM-NVM

Supplementary Note 2: Conformal contact FEM simulation

Supplementary Note 3: Programming energy calculation

Supplementary Note 4: Analysis of the adhesion force between metallic surfaces

Supplementary Note 5: An analytical stiffness model and operation design process

Supplementary Note 6: CMOS-compatible device fabrication on an 8-inch wafer

Supplementary Note 7: Analysis of the program operation

Supplementary Note 8: Estimation of the erase temperature

Supplementary Note 9: Stability of the NEM-NVM in harsh environments

References

Supplementary Note 1: Limitations of the conventional NEM-NVM

Supplementary Fig. 1a shows the structure and operation mechanisms of the conventional nanoelectromechanical non-volatile memory (NEM-NVM). It consists of a deformable beam and two in-plane electrodes for program and erase operations. For program operation, when an operating voltage is applied between the deformable beam and program electrode, the beam is deflected laterally by the electrostatic force generated from the program electrode and is held in contact by an adhesion force. In the case of the erase operation, similarly, the deformable beam is deflected in the opposite direction by the erase electrode. Supplementary Fig. 1b, c show cross-sectional transmission electron microscope (TEM) images of the initial state and the programmed state of the conventional NEM-NVM, respectively. This NEM-NVM was fabricated using a back-end-of-line process of the TSMC company, which is similar to the fabrication method used in previous studies[1,2]. The in-plane electrode configuration makes the fabrication process very easy and simple. Supplementary Fig. 1d shows the fabrication method of the conventional NEM-NVM with an in-plane electrode configuration. First, a sacrificial layer and structural material are sequentially deposited on the Si wafer, and then a deformable beam and two in-plane electrodes are patterned through a photolithography and an etching process. Finally, the sacrificial layer is removed to release the deformable beam.

The conventional NEM-NVM has several issues related to the energy-efficiency. The first problem is the in-plane electrode configuration. The air gap and width of the deformable beam, the most important factors of the operating energy, are determined by the resolution of the photolithography tools. Therefore, this limits the effective scaling of the operating energy. The second problem is the electrostatic erase operation. It inevitably doubles the actuation air gap distance, which causes an increase in the operating energy and voltage starting with the second operation. Therefore, the conventional NEM memory devices typically have a long beam shape to reduce the operating energy and voltage. Additionally, the inclined sidewall of the in-plane

electrode configuration is the main reason for performance degradation. As shown in Supplementary Fig. 1b, the side wall, where the contact occurs, is fabricated to be inclined due to the limitation of the patterning process. The inclined sidewall makes it difficult to implement conformal contact between the beam and operation electrodes. This non-conformal contact can cause performance degradation such as high contact resistance, low reliability, and reduced retention performance.

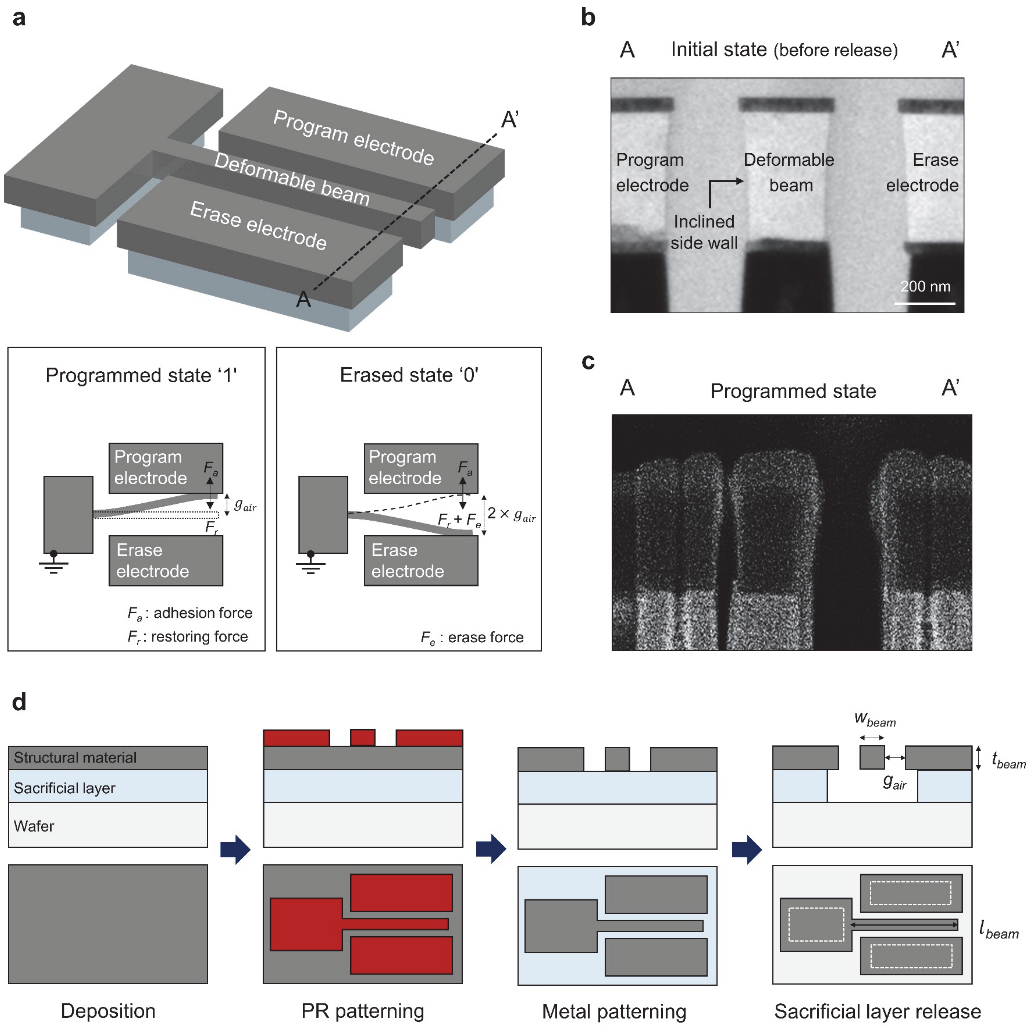

Supplementary Fig. 1 | Structure, operation mechanisms, and fabrication method of the conventional NEM-NVM. a Conventional NEM-NVM with an in-plane electrode configuration and its operation mechanisms. **b**,**c** Cross-sectional TEM images before the release process and after program operation, respectively. **d** Fabrication process of the conventional NEM-NVM with an in-plane electrode configuration.

Supplementary Note 2: Conformal contact FEM simulation

Unlike the conventional NEM-NVM, the proposed NEM-NVM does not have an inclined sidewall issue because the contacting surfaces are fabricated by a deposition process. Supplementary Fig. 2 shows a three-dimensional finite-element method (FEM) simulation result and extracted beam profile after program operation of the NEM-NVM. This result indicates that the conformal contact can be made at the contacting surfaces.

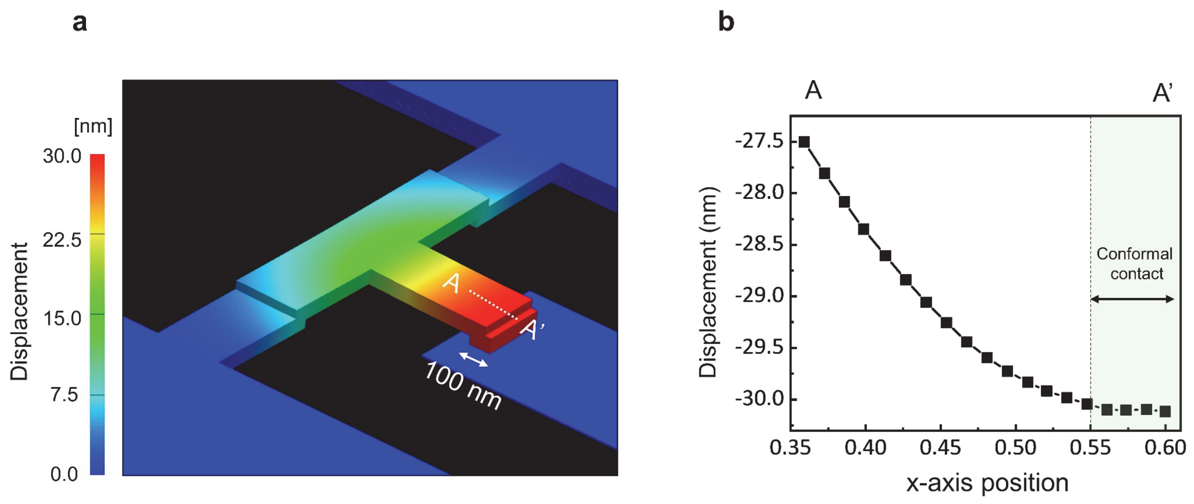

Supplementary Fig. 2 | Three-dimensional FEM simulation result of the program operation. a Displacement result of the NEM-NVM after program operation. **b** Extracted displacement profile of the bent cantilever.

Supplementary Note 3: Programming energy calculation

The energy consumed in the program operation of the NEM-NVM is calculated by the following equation[3].

$$E_{total} = E_{electrical} + E_{mechanical\,spring} + E_{kinetic} + E_{damping} \quad (1)$$

$$= \frac{1}{2}CV^2 + \frac{1}{2}kg_{air}^2 + \frac{1}{2}mv^2 + \frac{3\mu A^2}{2\pi g^3}\int v^2 dt \approx \frac{1}{2}CV^2 + \frac{1}{2}kg_{air}^2 \quad (2)$$

where C is the capacitance between the top and bottom electrodes, V is the operating voltage, k is the mechanical spring constant, g_{air} is the actuation air gap, m is the total mass of the top electrode, v is the velocity related to the movement of the top electrode, and μ is the viscosity of quasi-ideal gas at standard temperature and pressure. Since the kinetic energy and energy lost in damping are relatively very small, the electrical energy and mechanical spring energy are the dominant values in the total operation energy. The capacitance and operating voltage are calculated by the following equation[3]. Therefore, the total operation energy is determined by the actuation air gap and stiffness as shown in Fig. 1d.

$$C = \frac{\varepsilon_0 A}{g_{air}} \quad (3)$$

$$V^2 = \frac{8kg_{air}^3}{27\varepsilon_0 A} \quad (4)$$

Supplementary Note 4: **Analysis of the adhesion force between metallic surfaces**

Generally, van der Waals (vdW) force and metallic bonding force are the dominant forces among the various interfacial forces between rough surfaces. Therefore, separation distance (D_{sep}) and the actual contact area (a) between the tungsten surfaces are needed to calculate the adhesion force. The van der Waals force and metallic bonding force between rough surfaces are calculated by the following equations[4-7].

$$F_{vdW}(D_{avg}) = \frac{A}{6\pi \cdot D_{sep}^3} \cdot g_f(D_{sep}) \times Area \quad (5\text{-}1)$$

$$g_f(D_{sep}) = 1 - \frac{2 \cdot D_{sep}}{c} + \frac{6 \cdot D_{sep}^2}{c^2} + \frac{12 \cdot D_{sep}^3}{c^3} - \frac{12 \cdot D_{sep}^3}{c^4} \cdot (D_{sep} + c) \cdot \ln(1 + \frac{c}{D_{sep}}) \quad (5\text{-}2)$$

$$F_{m-m} = 2\pi \bar{R} W \ (for\ elastic\ defomration), \qquad \bar{R} = \frac{R_1 R_2}{R_1 + R_2} \quad (6\text{-}1)$$

$$F_{m-m} = H\pi a^2 \ \ (for\ plastic\ defomration) \quad (6\text{-}2)$$

where A is the hamarker constant, g_f is function representing the retardation of F_{vdW} with separation distance, c is the characteristic wavelength constant, R is the radius of curvature of asperities, W is the work of adhesion, and H is the hardness. However, it is very challenging to accurately measure the separation distance and the actual contact area because the contact surfaces are buried in the two bodies. Thus, we used the well-established adhesion model[4] that can simulate the actual contact process using real roughness data to analyze the adhesion force. First, we measured the roughness of the surfaces having the same thickness as the top and bottom electrodes of the NEM-NVM. The scan size was 1×1 μm^2 with 256 pixels (approximately 4 nm lateral resolution). And then, the randomly extracted data (about 300 nm^2 area) from the measured surface roughness results were imported into the adhesion model that can analyze the separation distance and the actual contact area. As a result, the final adhesion force between the tungsten surfaces considering both van der Waals force and metallic bonding

force was determined to be 0.96 μN.

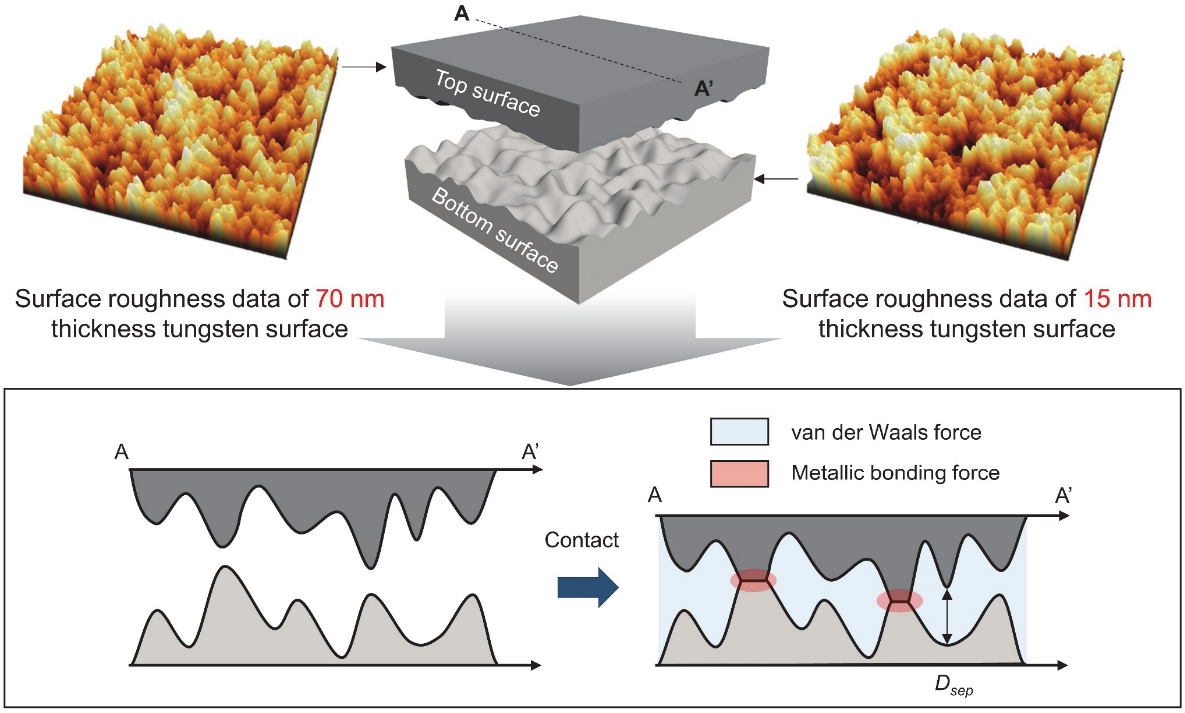

Supplementary Fig. 3 | Analysis process of the adhesion force between the metallic surfaces. The surface roughness of the two samples corresponding to the top electrode and the bottom electrode were measured, and then the adhesion force between the metallic surfaces was analyzed through the adhesion model considering the van der Waals force and the metallic bonding force.

Supplementary Note 5: An analytical stiffness model and operation design process

Precise design of the mechanical structure is very important for reliable operation of the NEM-NVM, which stores information through mechanical movement. Therefore, we used both Castigliano's theorem and FEM simulation to model the mechanical structure, and analyzed the parameters for reliable program and erase operations (Supplementary Fig. 4, 5). Finally, the optimally designed NEM-NVM through the mechanical analysis is shown in Supplementary Fig. 6.

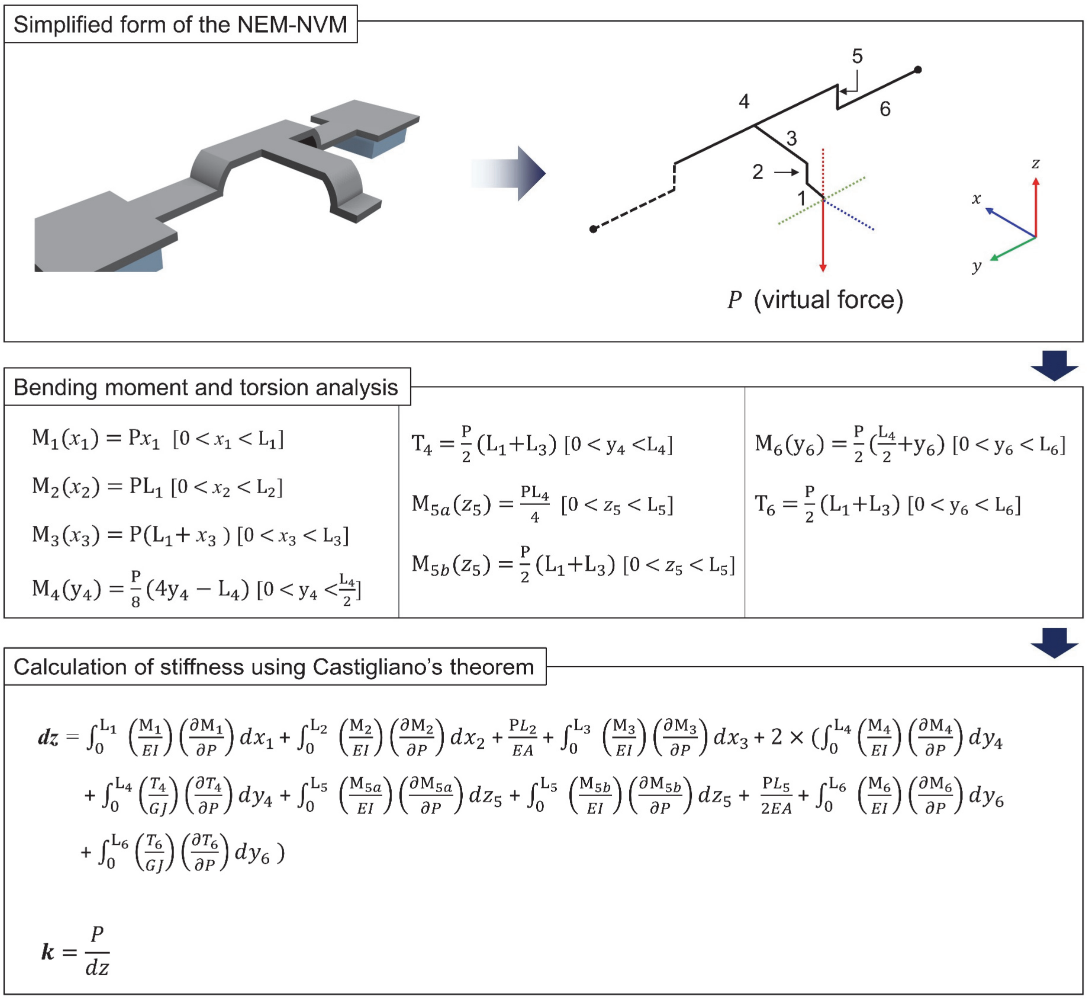

Supplementary Fig. 4 | Analytical stiffness model of the NEM-NVM. Each part of the top electrode was simplified, and the bending moments and torsions by the virtual force were found. Through Castigliano's theorem, the displacement at the end of the bent cantilever was calculated. The stiffness is the virtual force divided by the displacement.

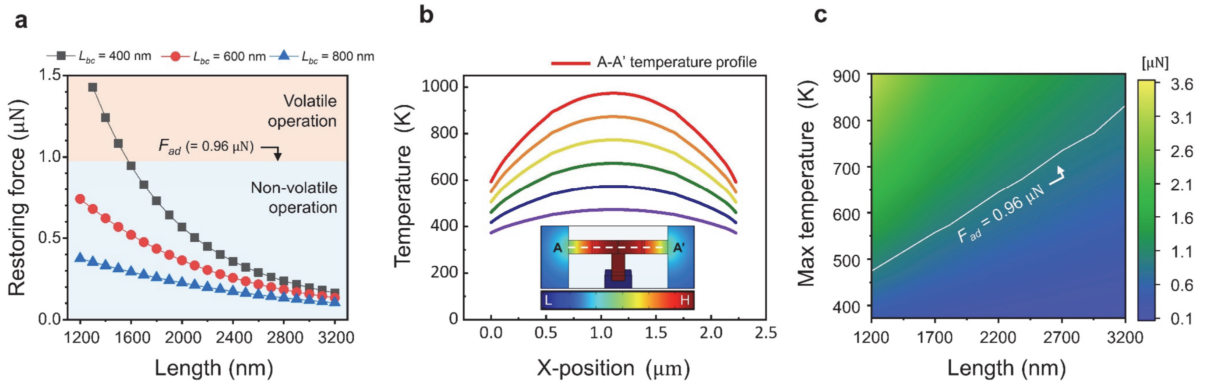

Supplementary Fig. 5 | Determining the dimensions for stable program and erase operations. a Calculated restoring force with respect to the length of the bent cantilever and pipe-clip spring (air gap, g_{air} = 30 nm; thickness, t_{beam} = 70 nm). **b** Three-dimensional resistive heating simulation result and derived temperature profiles of the pipe-clip spring. **c** Calculated thermal expansion force with respect to the length of pipe-clip spring and operating temperature.

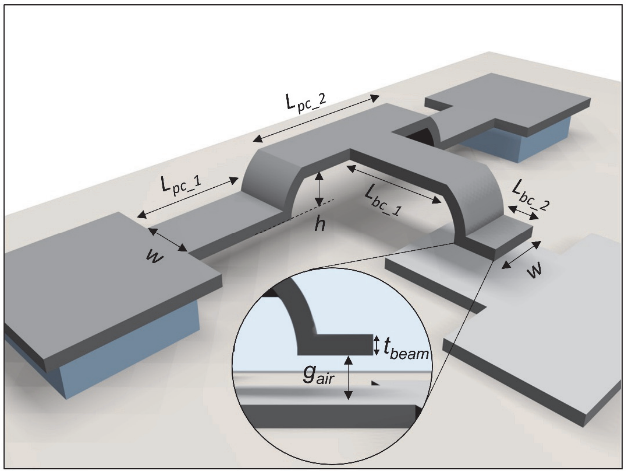

Parameters	Symbol	Value (nm)
Width	w	300
Thickness	t_{beam}	70
Initial air gap	g_{air}	30
Step height	h	40
Pipe-clip spring	L_{pc_1}	550
	L_{pc_2}	1100
Bent cantilever	L_{bc_1}	500
	L_{bc_2}	100

Supplementary Fig. 6 | Dimension information of the optimally designed NEM-NVM.

Supplementary Note 6: CMOS-compatible device fabrication on an 8-inch wafer

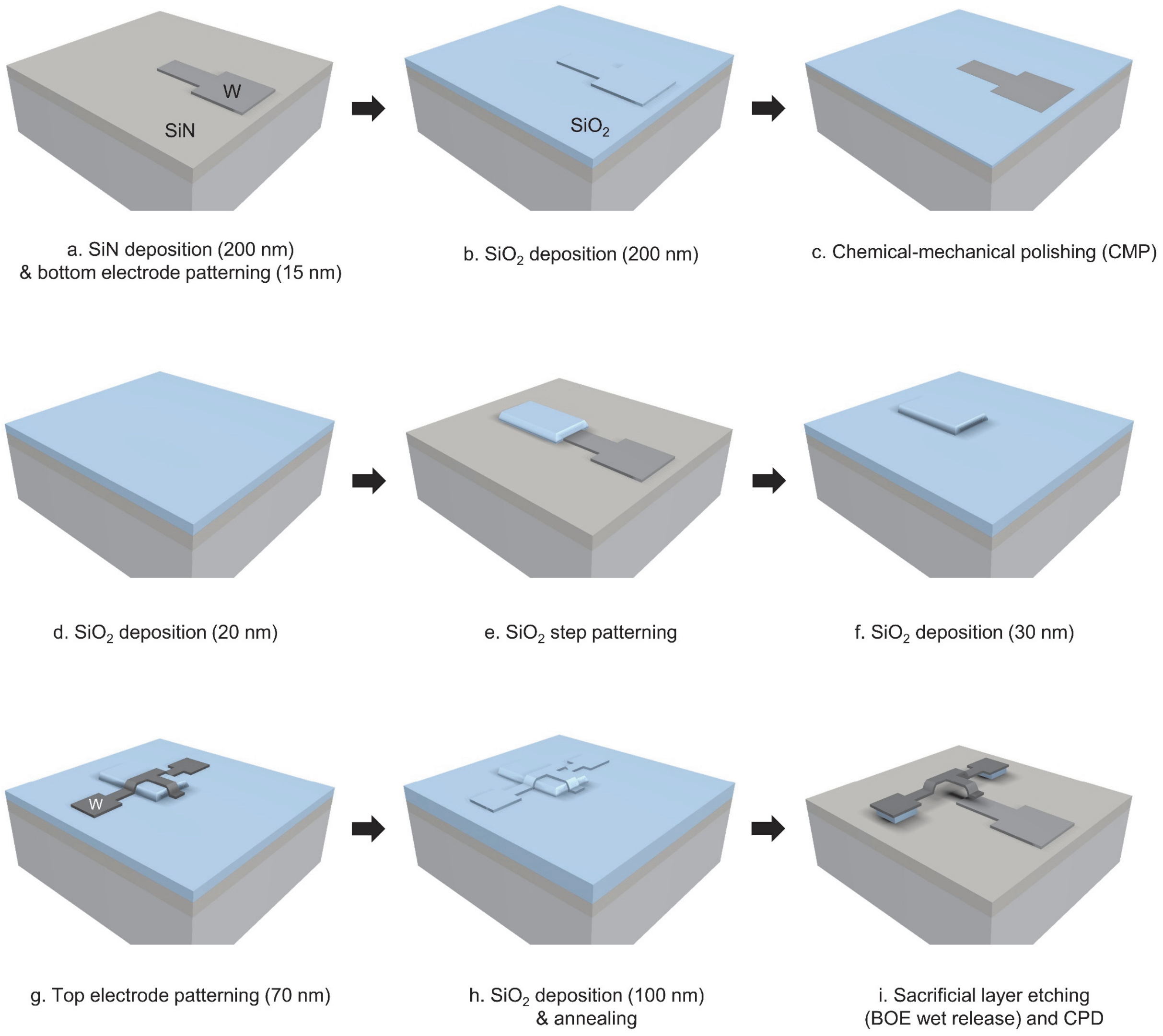

Supplementary Fig. 7 | CMOS-compatible device fabrication using the well-established manufacturing process. a Silicon nitride deposition (200 nm) and bottom electrode formation (tungsten, 15 nm). **b** 1st silicon dioxide deposition (200 nm). **c** Chemical-mechanical polishing process. **d** 2nd silicon dioxide deposition (20 nm). **e** Silicon dioxide patterning. **f** 3rd silicon dioxide deposition (30 nm). **g** Top electrode formation (tungsten, 70 nm). **h** 4th silicon dioxide deposition (100 nm) and annealing process (500 ℃, 30 min). **i** Sacrificial silicon dioxide etching using a buffered oxide etchant (BOE) solution and critical point drying process.

Supplementary Note 7: Analysis of the program operation

We need to know the actual air gap and the capacitance in the initial state to accurately calculate the programming energy. Through the measured operating voltage (9.9 V) as shown in Fig. 3a, the actual air gap was estimated using an analytical stiffness model (from Supplementary Note 5) and FEM simulation, respectively (Supplementary Fig. 8a). Both methods show that the actual air gap of the NEM-NEM is about 20 nm. Through the estimated actual air gap, the capacitance between the top and bottom electrodes was calculated (C =16.46 aF). As a result, the programming energy of the fabricated NEM-NVM was calculated by substituting the capacitance, operation voltage, actual air gap, and stiffness (10.15 $\mathrm{N\ m^{-1}}$) into the following equation.

$$E_{total} \approx E_{electrical} + E_{mechanical\,spring} = \frac{1}{2}CV^2 + \frac{1}{2}kg_{air}^2 = 2.83 \ \mathrm{fJ\ bit^{-1}}$$

We also measured the I-V dual sweep curves of the NEM-NVM as shown in Supplementary Fig. 8b. When the applied voltage was increased above the threshold (program) voltage and then swept back to zero, 'On' current maintained the compliance value of 100 nA, but gradually decreased as the applied voltage decreased. These results indicate that the NEM-NVM successfully operated in a non-volatile manner via delicate engineering of the mechanical structure as we described in Supplementary Note 5. Furthermore, due to the reliable fabrication using the CMOS manufacturing processes, the memory devices also show very uniform contact characteristics.

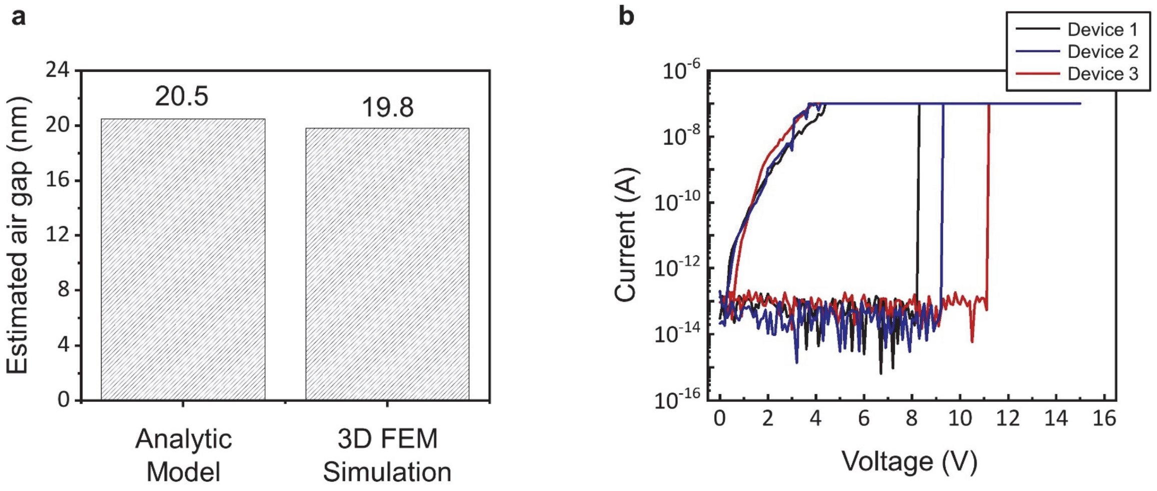

Supplementary Fig. 8 | Evaluation of the program operation. a Estimated actuation air gap through the analytical stiffness model and 3D FEM simulation. **b** I-V Dual sweep curves of the three NEM-NVM devices, showing very uniform contact characteristics

Supplementary Note 8: Estimation of the erase temperature

A resistance-temperature detector (RTD) method and time-dependent FEM simulation were conducted to estimate the erase temperature of the NEM-NVM. First, the resistance of the pipe-clip spring was measured according to the temperature on an external hotplate. The exact temperature of the pipe-clip spring on the heated hotplate was measured by an infrared thermometer. As shown in Supplementary Fig. 9a, the resistance of the pipe-clip spring has a linear correlation with the temperature due to the stable thermal property of tungsten[8]. The extracted thermal coefficient of resistance (TCR) was 1750 ppm K^{-1}. Using this result, we could predict the maximum temperature (1000.4 K) at the erase voltage (0.9 V). However, since this value is evaluated at a bulk time scale, the time-dependent FEM simulation should be supplemented to determine the temperature at 400 ns. Supplementary Fig. 9b shows the temperature of the pipe-clip spring with respect to the time, under the condition that the final temperature reaches 1000.4 K through resistive heating. The temperature at 400 ns was 873.1 K, which is the maximum temperature of the erase operation (the average temperature was 776.4 K)

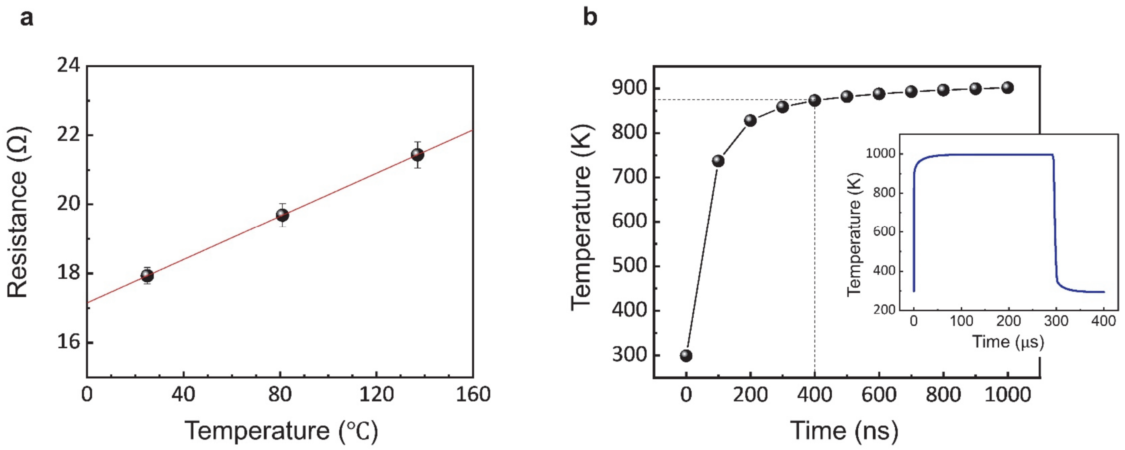

Supplementary Fig. 9 | Evaluation of the erase temperature. a Measured resistance of the pipe-clip spring with respect to the temperature on an external hot plate. The error bars represent the standard deviations among the pipe-clip spring ($n \geq 8$). **b** Time-dependent resistive heating simulation result.

Supplementary Note 9: Stability of the NEM-NVM in harsh environments

Thanks to the radiation-robust structural material and mechanically operating mechanisms, the NEM-NVM shows excellent radiation-hardness without additional rad-hard processing and complex circuit configurations. The electrical conductivity of nano-patterned tungsten and I-V characteristics of the NEM-NVM before and after exposure to the high-energy radiation was shown in Supplementary Fig. 10, 11, respectively. The NEM-NVM also demonstrated superb stability of stored information against shock, vibration and high temperature. The mechanical features and design process of the NEM-NVM enabling the high stability of the mechanical bits in harsh environments are shown in Supplementary Fig. 12, 13.

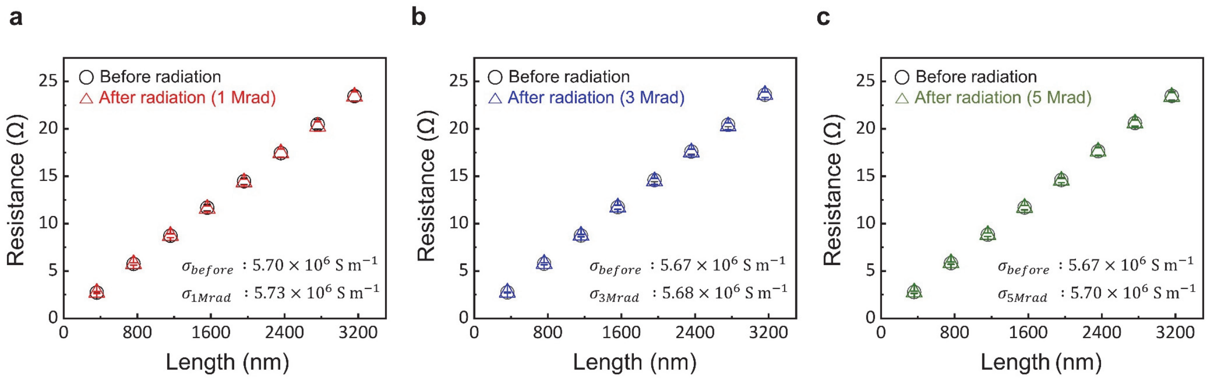

Supplementary Fig. 10 | Resistance of nano-patterned tungsten before and after exposed to radiation. Comparison of the resistance of the fixed-fixed tungsten beams before and after the gamma-ray irradiation: **a** 1 Mrad. **b** 3 Mrad. **c** 5 Mrad. The error bars represent the standard deviations among the beams ($n \geq 6$).

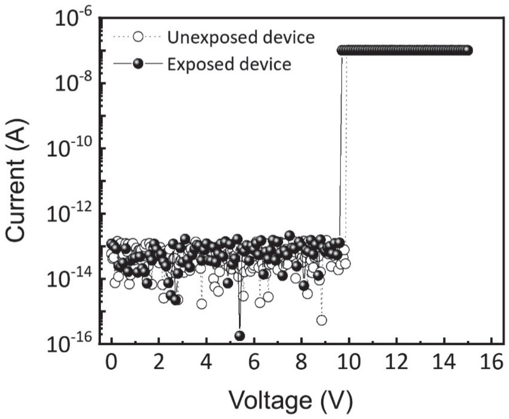

Supplementary Fig. 11 | I-V characteristics of the NEM-NVM before and after exposed to radiation. The NEM-NVM retained its operation characteristics even after 1 Mrad gamma-ray irradiation (dashed curve is the same I-V characteristics as shown in Fig. 3a).

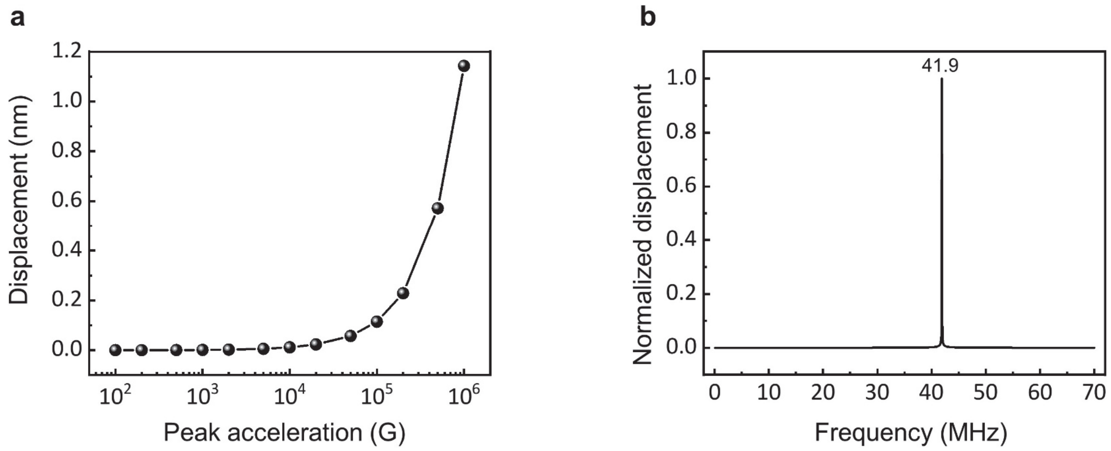

Supplementary Fig. 12 | Robustness of the NEM-NVM to mechanical stimuli. a Calculated displacement of the NEM-NVM with respect to the acceleration. **b** Frequency domain simulation result of the NEM-NVM.

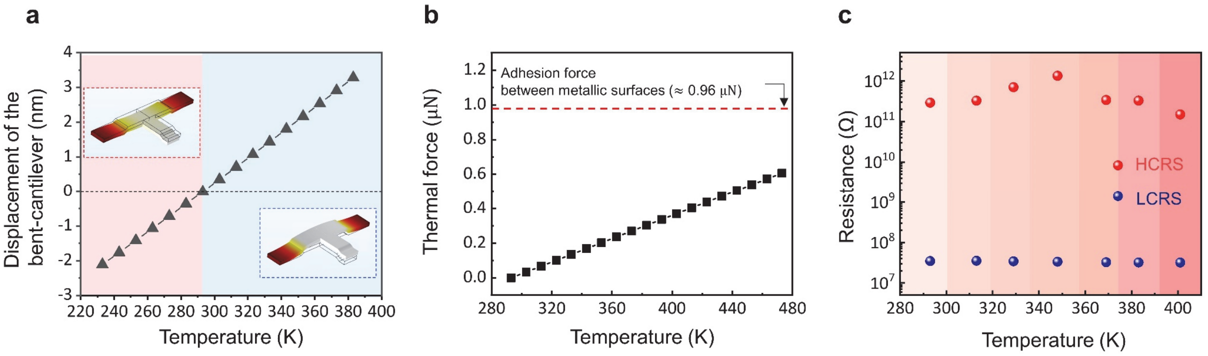

Supplementary Fig. 13 | Stability of the NEM-NVM over a wide temperature range. a Ambient temperature-induced displacement of the NEM-NVM. **b** Ambient temperature-induced thermal force of the pipe-clip spring. **c** Mechanical bit stability at high temperature up to 400 K.

References

1. Kwon, H. S., Kim, S. K. & Choi, W. Y. Monolithic three-dimensional 65-nm CMOS-nanoelectromechanical reconfigurable logic for sub-1.2-V operation. *IEEE Electron Device Letters* **38**, 1317-1320 (2017).
2. Sikder, U. *et al.* Toward Monolithically Integrated Hybrid CMOS-NEM Circuits. *IEEE Trans. Electron. Devices* **68**, 6430-6436 (2021).
3. Rebeiz, G. M. *RF MEMS: Theory, Design, and Technology* (John Wiley & Sons, 2004).
4. DelRio, F. W. *et al.* The role of van der Waals forces in adhesion of micromachined surfaces. *Nature materials* **4**, 629-634 (2005).
5. Anandarajah, A. & Chen, J. Single correction function for computing retarded van der Waals attraction. *Journal of colloid and interface science* **176**, 293-300 (1995).
6. Johnson, K. Mechanics of adhesion. *Tribology International* **31**, 413-418 (1998).
7. Smith, C. G. *et al.* in *MEMS/MOEMS Components and Their Applications IV.* 43-50 (SPIE).
8. Cezairliyan, A. & McClure, J. High-speed (subsecond) measurement of heat capacity, electrical resistivity, and thermal radiation properties of tungsten in the range 2000 to 3600 K. *Journal of Research of the National Bureau of Standards. Section A, Physics and Chemistry* **75**, 283 (1971).
